# A Closed System for Pico-Liter Order Substance Transport from a Giant Liposome to a Cell

**DOI:** 10.3390/mi9070331

**Published:** 2018-07-02

**Authors:** Shohei Miyakawa, Kaoru Uesugi, Keisuke Morishima

**Affiliations:** Department of Mechanical Engineering Graduate School of Engineering, Osaka University, 2-1 Yamadaoka, Suita, Osaka 565-0871, Japan; shohei.miyakawa@gmail.com (S.M.); uesugi@live.mech.eng.osaka-u.ac.jp (K.U.)

**Keywords:** microfluidic device, nano-liter order fluidic device, pico-liter order fluidic device, µ-TAS, giant liposome, single cell, electrofusion, substance transport

## Abstract

In single cell analysis, transport of foreign substances into a cell is an important technique. In particular, for accurate analysis, a method to transport a small amount (pico-liter order) of substance into the cell without leakage while retaining the cell shape is essential. Because the fusion of the cell and the giant liposome is a closed system to the outside, it may be possible to transport a precise, small amount of substances into the cell. Additionally, there is no possibility that a leaked substance would affect other systems. To develop the liposome-cell transportation system, knowledge about the behavior of substances in the liposome and the cell is important. However, only a few studies have observed the substance transport between a liposome and a cell. Here, we report observation of small amount of substance transport into a single C2C12 cell by using a giant liposome. Substance transport occurred by electrofusion between the cell and the giant liposome containing the substance, which is a closed system. First, to observe the electrofusion and substance transport from the moment of voltage application, we fabricated a microfluidic device equipped with electrodes. We introduced suspensions of cells and liposomes into the microfluidic device and applied alternating current (AC) and direct current (DC) voltages for electrofusion. We observed a small amount (22.4 ± 0.1%, 10.3 ± 0.4% and 9.1 ± 0.1%) of fluorescent substance (Calcein) contained in the liposomes was transported into the cell without leakage outside the cell, and we obtained the diffusion coefficient of Calcein in the cell as 137 ± 18 μm^2^/s. We anticipate that this system and the knowledge acquired will contribute to future realization of more accurate single cell analysis in a wide range of fields.

## 1. Introduction

Cells are the smallest unit of living things. However, many things are unknown in regard to cells, so that analyses on a single cell scale are very meaningful in the fields of biology and medicine. Transporting substances into a cell and observing the cell behavior has been widely used as a cell analysis method suited to various purposes. Gene transfer into a cell by lipofection or an electroporation method is a representative example [1,2,3]. By analyzing gene expression, a functional analysis of genes becomes possible. These functional analysis methods are conducted by chemical or electrical treatment of the cell and floating the substances for transport in a dish or micro channel and chamber [4,5,6,7,8,9].

However, these methods cannot precisely transport small amounts of substances because it is inevitable that substances adhere to the channel wall. Moreover, since it is necessary to suspend a large amount of substances in a volume that is larger than the volume of the cell, they are unsuitable for transport of rare substances.

In consideration of these problems, a method is required to transport a precise amount of substance on a single cell scale for accurate analysis. If a substance is encapsulated in a micro capsule for which the diameter can be adjusted and the substrate is transported from the micro capsule to a cell by membrane fusion between them, a precise amount of substance can be transported to the cell. As a tool that can realize such a method, giant liposomes may be a good candidate. Giant liposomes are vesicles having a closed lipid bilayer structure in a spherical shell shape, encapsulating substances and their diameter is more than 10 μm [10,11,12]. By fusing a cell with a giant liposome containing transport substances in the inner liquid layer, it becomes possible to transport substances into the cell [13,14]. Because the fusion of the cell and the giant liposome is a closed system to the outside, it is possible to transport a precise, small amount of substances into the cell. Additionally, there is no possibility that a leaked substance would not affect other systems. Moreover, since it is possible to control the content of the giant liposome by changing the fabrication protocol, the giant liposome becomes a substance transport capsule that can accurately transport a desired amount of substances into the cell. The final benefits of the fusion method are its lower invasiveness and higher efficiency than microinjection.

Previously, Saito et al. [14] conducted electrofusion between a cell and a giant liposome and introduced microbeads and “DNA origami” made of actual folded DNA into a cell. However, they did not observe the moment of electrofusion in their study. Since cells and liposomes are freely moved by Coulomb force in an ordinary millimeter-scale electrofusion chamber, it is difficult to identify observation targets and align a microscope. To evaluate the reaction of the transported substance inside the cell and the transport speed, observation from the moment of fusion is essential. Shirakashi et al. [13] observed electrofusion between a cell and a giant liposome at a fixed point using dielectrophoretic force generated between two stainless steel wires.

However, there have been only a few studies which observed the substance transport between a liposome and a cell in detail. In studying electrofusion between a cell and a liposome, it is necessary to observe the transport of substances into the cell that retains its shape from the moment of electrofusion for dynamic observation of the response inside the single cell to the transported substances.

In this paper, we report on electrofusion and a small amount (pico-liter order) of substance transport between a cell that retains its shape and a giant liposome; this is a closed system. Because the volume of the liposome-containing substance is pico-liter order (diameter of the liposome is several tens of micrometers), the volume of the transported substrate is also pico-liter order. We can apply the proposed method to develop a micro-, nano- or pico-liter order fluidic device which can control the amount of substances accurately. First, we present the principle and describe the fabrication of the microfluidic device for electrofusion observation between a cell and a liposome in details. Then, we present observation results and image analysis of the substance transport between the cell and the liposome. Only a few studies have observed the transport of substance between a liposome and a cell. Thus, the transport observation of this study will be useful for research on liposomes.

## 2. Materials and Methods

### 2.1. Fabrication of the Microfluidic Device and Optimization of Suspension Concentrations

**Figure 1:**
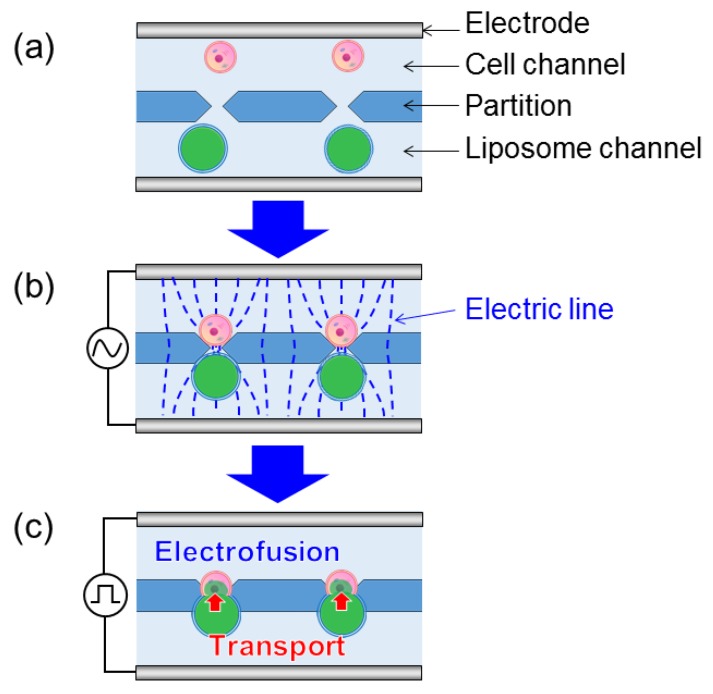
Principle of the one-to-one electrofusion method between a cell and a giant liposome. (**a**) Cells and giant liposomes were separately introduced into the partitioned microchannel. (**b**) An alternating current (AC) voltage was applied between the electrodes. Cells and giant liposomes approached gaps between the partitions due to positive dielectrophoretic force and they adhered to each other by Coulomb force. (**c**) A direct current (DC) voltage was applied. Electrofusion and transport of substance occurred between the cell and the giant liposome.

We adopted the one-to-one electrofusion method developed by Masuda et al. [15] to observe substance transport between a cell and a giant liposome (Figure 1). Cells and giant liposomes were separately introduced into one side of the partitioned microchannel (Figure 1a). Their introduction was done by capillary forces occurring in the microchannel. By applying an alternating current (AC) voltage between the flat electrodes, cells and giant liposomes approached the gaps between the partitions due to positive dielectrophoretic force and they adhered to each other by Coulomb force (Figure 1b). Next, by applying a direct current (DC) voltage between the flat electrodes, membrane breakdown and membrane fusion occurred at the adhesion point between the cell and the giant liposome, and the substance contained in the liposome was transported into the cell (Figure 1c).

**Figure 2:**
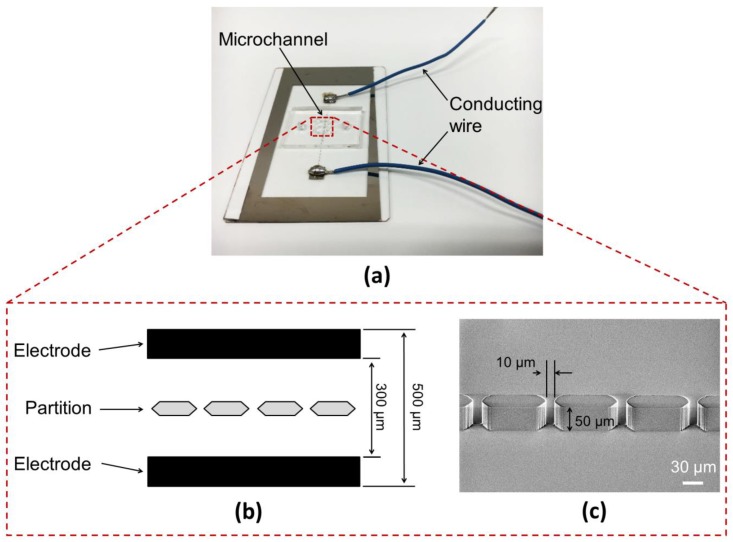
(**a**) Photo of the fabricated microfluidic device; (**b**) schematic illustration of the partitions and the electrodes; and (**c**) Scanning electron microscope (SEM) image of the partitions.

The fabricated microfluidic device consisted of two parts: a flat electrode substrate and poly(dimethylsiloxane) (PDMS) microchannel (Figure 2a). These were fabricated by photolithography technology. At first, to fabricate the flat electrode substrate, a 100 nm Ni layer was sputtered on a slide glass (Matsunami S9111) using a sputtering apparatus (CFS-4ES-SS, Shibaura, Tokyo, Japan). Positive photoresist (OFPR-800LB, Tokyo Ohka Kogyo, Kawasaki, Japan) was coated on the Ni layer. Photoresist in the area excluding the electrode pattern was removed by ultraviolet (UV) irradiation (Mask Aligner: MA-10, Mikasa Co., Ltd., Tokyo, Japan) and the exposed Ni layer was etched to form an electrode pattern. The distance between the electrodes was 300 µm and the electrodes were on either side of the microchannel space which was 500 µm wide (Figure 2b). The microchannel was fabricated by transferring channel patterns to PDMS (SILPOT 184, Dow Corning, Midland, MI, USA) using a mold. The gap between partitions and the partition height were 10 µm and 50 µm, respectively (Figure 2c), and these values were selected based on the diameters of a cell and a giant liposome. Because the diameters of a cell (about 20 µm) and a giant liposome (about 40 µm) were larger than the gap between the partitions, mixing of cells and giant liposome was prevented. Thus, 1:1 pairs were formed effectively. The number of gaps was 76.

**Figure 3:**
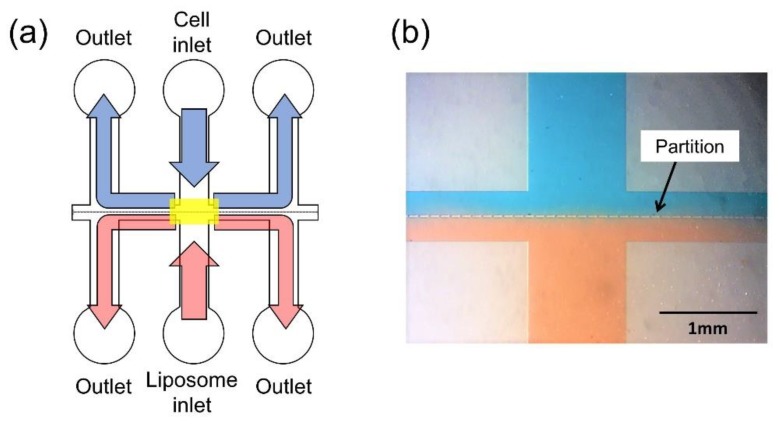
(**a**) The flow of cells and liposomes in the microchannel by capillary force. The introduced suspensions of cells and liposomes flowed towards each of the two outlets on both sides of the inlet. (**b**) Bright field image of red and blue solutions that were introduced in the microchannel at the yellow rectangle region of (**a**).

Cells and giant liposomes were trapped in the gaps between partitions of the microchannel. The PDMS microchannel surface was made hydrophilic by plasma ion irradiation to enable introduction of cells and giant liposomes by capillary force and the PDMS microchannel was bonded to the electrode substrate. The flow of the suspensions of cells and liposomes in the microchannel is shown in Figure 3a. Red and blue solutions were introduced into the microchannel for visualization of the flow and the bright field image of the yellow rectangle region in Figure 3a is shown in Figure 3b.

### 2.2. Optimization of Suspension Concentrations

We optimized concentrations of the suspensions for highly efficient pair formation in the microchannel. A cell suspension was prepared by dissolving C2C12 cells (mouse myoblast cells) (Riken Cell Bank) into the fusion buffer. To carry out the observation simply, we used only cells because it took a long time and a complex process was needed for preparing liposomes. The fusion buffer consisted of 310 mM mannitol (133-00845, Wako Pure Chemical Industries, Osaka, Japan), 0.1 mM MgCl_2_ (135-00165, Wako Pure Chemical Industries), and 0.1 mM CaCl_2_ (10043-52-4, Wako Pure Chemical Industries), mixed into pure water as solvent. The cell suspension buffer was introduced into the microchannel and the number of pairs formed in the microchannel by applying the AC voltage (6 V, 1 MHz, 20 s) using the electrofusion device (ECFG21, Nepa Gene, Ichikawa, Japan) was measured. The concentration of the cell suspension was changed from 10^3^ cells/mL to 10^5^ cells/mL. In addition, 20 µL of each suspension of C2C12 cells (the range of 10^3^ cells/mL to 10^5^ cells/mL) was fluorescently labeled in two colors with CellTracker Red and Green (C34552 and C7025, Thermo Fisher Scientific, Waltham, MA, USA). For recognizing cells introduced from the upper side inlet and cells introduced from the lower side inlet, we dyed cells introduced for each side inlet using different colors. Then, each cell suspension was introduced into the respective inlets of the microchannel (“cell inlet” and “liposome inlet”) and observed with a fluorescence microscope (BZ-X700, Keyence, Osaka, Japan). The experimental observations were carried out three times.

### 2.3. Preparation of Cells

C2C12 cells were prepared for the observation of pair formation and the substance transport experiment. The cells were cultured in an incubator maintained at 37 °C and 5% CO_2_. The culture medium was Dulbecco’s Modified Eagle’s Medium (08458-16, DMEM, Nacalai Tesque, Kyoto, Japan) supplemented with 10% fetal bovine serum (172012, Sigma-Aldrich, St. Louis, MO, USA), 100 units/mL penicillin and 100 µg/mL streptomycin (P4333, Sigma-Aldrich).

### 2.4. Preparation of Giant Liposomes

Giant liposomes were prepared using the water-in-oil emulsion centrifugation method [10,14], with modification. The lipid solution was prepared by dissolving 45 mg of dioleoylphosphatidylcholine (MC-8181, DOPC, NOF Corp., Tokyo, Japan), 5 mg of dioleoylphosphatidylglycerol (MG-8181LS, DOPG, NOF Corp.), and 2.5 mg of cholesterol (034-03002, Wako Pure Chemical Industries) in 525 µL of chloroform (035-02616, Wako Pure Chemical Industries). The solution was poured into a tube and dried under vacuum for 3 h. Then 250 µL of liquid paraffin (26137-85, Nacalai Tesque) was poured into the tube and treated by ultrasonication at 65 °C for 60 min. To prepare an emulsion suspension, 25 µL of an inner solution was poured into the microtube and mixed with a mixer (VORTEX-GENIE 2, Scientific Industries, Inc., New York, NY, USA). The inner solution consisted of 30 mM sucrose (196-00015, Wako Pure Chemical Industries), 70 mM mannitol, 0.1 mM MgCl_2_, 0.1 mM CaCl_2_, 10 mM NaOH (37847-08, Kanto Chemical Industry, Tokyo, Japan), and 1 µM Calcein (prepared by treating Calcein-AM (341-07901, Dojindo Laboratories, Kumamoto, Japan) with alkali solution), mixed with pure water as solvent. Then, 125 µL of the emulsion suspension was poured into 1 mL of outer solution in the microtube and centrifuged at 18,000× *g*, 4 °C, 30 min. The outer solution consisted of 110 mM mannitol, 0.1 mM MgCl_2_, and 0.1 mM CaCl_2_, mixed with pure water as solvent.

### 2.5. Observation of Electrofusion and Substance Transport between the Cell and the Giant Liposome

**Figure 4:**
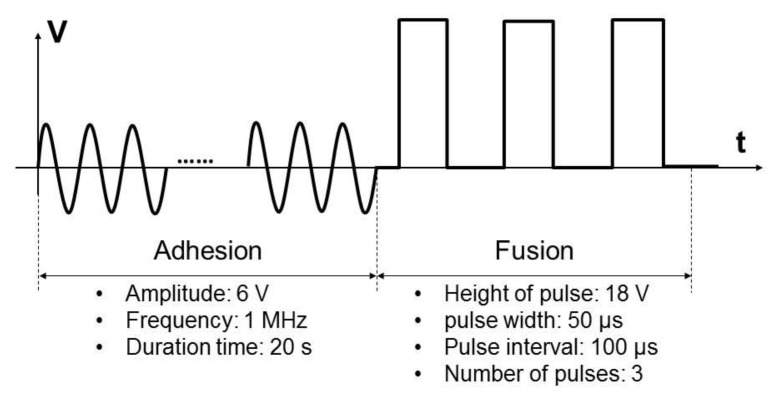
Schematic diagram of the electrical stimulation pulses for fusion between cells and giant liposomes. After introducing the suspension in the microchannel, first an AC voltage (6 V; 1 MHz; 20 s) and then a DC voltage (18 V; pulse width, 50 μs; pulse interval, 100 μs; number of pulses, 3) were consecutively applied.

A 20 μL suspension of non-fluorescently labeled cells and giant liposomes in the fusion buffer (110 mM mannitol, 0.1 mM MgCl_2_, and 0.1 mM CaCl_2_, mixed in pure water as solvent) was prepared at a concentration of 10^4^ cells/mL and this was introduced into the microfluidic device. The cells were detached from the culture dish with 0.25% trypsin-EDTA solution (T4049, Sigma-Aldrich) and rinsed twice with DPBS (11482-15, Nacalai Tesque). After introducing the suspension, an AC voltage (6 V; 1 MHz; 20 s) and a DC voltage (18 V; pulse width, 50 μs; pulse interval, 100 μs; number of pulses, 3) were consecutively applied (Figure 4). Electrofusion and substance transport between a cell and a giant liposome were observed using the fluorescence microscope. Measured intracellular luminance was normalized with background luminance. Voltage application and observation were conducted on 50 pairs of a cell and a liposome. Using image analysis (Image J), we measured the percentage of transported substance amount, the time constant and the diffusion coefficient of intracellular luminance transition. The percentages (*R_A_*) were calculated by Equation (1):

(1) $$R_{A}=\frac{I_{C}}{I_{L}+I_{C}}$$

where *I_L_* and *I_C_* are plateau intensity of liposome and cell after transport of Calcein from the liposome to the cell by electrofusion.

In the measurement of the time constant, the time from the start of the increase in luminance to reach 63.2% of the steady-state value was calculated. The value of 63.2% was calculated by “(1 − 1/e)” (e = 2.71828: logarithm natural) which is obtained by the general theory of transient response. The diffusion coefficient was calculated by fitting the theoretical curve of the equation to the measurement value of intracellular luminance measured at intervals of 5 μm in the vertical direction to the fusion part of the cell and the liposome, which was defined as the origin (0 μm). The theoretical equation of concentration *c* in the cell can be expressed as Equation (2).

(2) $$c=\frac{c_{0}}{2}\left\{1+\mathrm{erf}\left(\frac{y}{2\sqrt{Dt}}\right)\right\}$$

where *y* is distance from the fusion part, *D* is the diffusion coefficient, *t* is the diffusion time, and *c*_0_ is concentration at the fusion part after *t* s [16].

## 3. Results and Discussion

### 3.1. Relationship between Suspension Concentration and Pair Formation Efficiency

**Figure 5:**
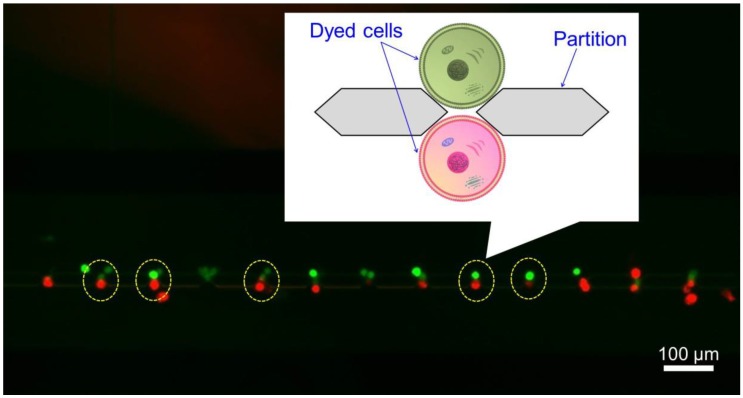
Fluorescent image of pairs of green and red dyed cells. Pairs with different colors were formed at the gaps between the partitions.

**Figure 6:**
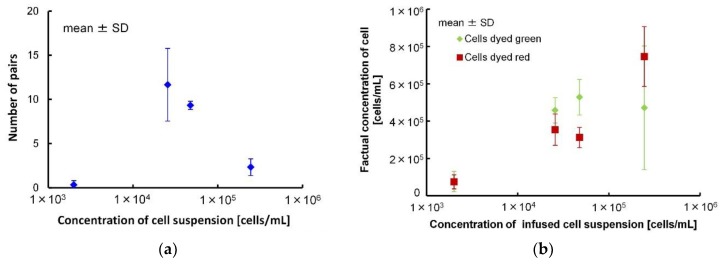
(**a**) Relationship between concentration of cell suspension and number of cell pairs formed in the microchannel by applying an AC voltage. (**b**) Relationship between concentration of cell and liposome suspension and concentration of introduced cells.

Yellow dotted circle of Figure 5 shows the pairs of green and red dyed cells. The suspension of red dyed cells was introduced from the upper side inlet and the suspension of green dyed cells was introduced from the lower side inlet. The relationship between the average of the suspension concentration and the measured number of pairs with different colors (green and red dyed cells) formed through the partitions in the microchannel is shown in Figure 6a. Pairs of multiple cells were excluded. The numbers of pairs were 0.3 ± 0.4 (2.00 × 10^3^ cells/mL), 11.7 ± 4.1 (2.57 × 10^4^ cells/mL), 9.3 ± 0.5% (4.76 × 10^4^ cells/mL) and 2.3 ± 0.9 (2.45 × 10^5^ cells/mL) (mean ± SD). The value of the efficiency of docking were derived by the dividing the number of pairs by the number of gaps (76). The values of the efficiency of docking were 0.4 ± 0.6% (2.00 × 10^3^ cells/mL), 15.4 ± 5.4% (2.57 × 10^4^ cells/mL), 12.3 ± 0.6% (4.76 × 10^4^ cells/mL) and 3.1 ± 5.4% (2.45 × 10^5^ cells/mL) (mean ± SD). When the concentration was 10^3^ cells/mL, the number of formed pairs was small because only a few cells were introduced into the microchannel. On the other hand, when concentration was 10^5^ cells/mL, only a few 1:1 pairs were formed because the number of cells introduced was large, and for example, 1:2 or 1:3 pairs were formed. As a result, while many of the introduced cells were deposited at the inlet, we found that the optimum suspension concentration was 10^4^ cells/mL. For either suspension concentration, 10^3^ cells/mL or 10^5^ cells/mL, since the capillary force occurred within the microchannel just after introduction, we did not observe that cells flowed to the microchannel later from the device inlet after pair formation. Therefore, when DC voltage was applied, fusion with cells or giant liposomes other than the target could be prevented.

Because the concentrations of the suspension were counted before introduction into the microchannel, there was a possibility that the concentrations of the suspension actually introduced into the microchannel were different from those before introduction (that is, before reaching the microchannel). Therefore, we counted the number of introduced cells and calculated the concentrations of suspension introduced into the microchannel. Figure 6b shows the concentrations which were introduced into the microchannel. The number of pairs could be affected by there being a smaller concentration of introduced suspension. The concentrations of the introduced suspension of red dyed cells in the 2.57 × 10^4^ cells/mL suspension (350 ± 80 cells/µL) and 4.76 × 10^4^ cells/mL suspension (310 ± 50 cells/µL) were smaller than concentrations for those same numbers of green dyed cells (2.57 × 10^4^ cells/mL; 460 ± 70 cells/µL, 4.76 × 10^4^ cells/mL; 530 ± 100). Additionally, the average concentration of the introduced suspension of red dyed cells of the 2.57 × 10^4^ cells/mL suspension (350 ± 80 cells/µL) was larger than that of the 4.76 × 10^4^ cells/mL suspension (310 ± 50 cells/µL). This indicated that the possible maximum number of pairs of the 2.57 × 10^4^ cells/mL suspension was larger than that of the 4.76 × 10^4^ cells/mL suspension. Thus, the number of pairs of the 2.57 × 10^4^ cells/mL suspension could be larger than that of the 4.76 × 10^4^ cells/mL suspension. The average concentration of introduced green and red dyed cells of the 2.45 × 10^5^ cells/mL suspension (470 ± 330 cells/µL) was closer to that of the other suspensions. On the other hand, the error of the 2.45 × 10^5^ cells/mL suspension was remarkably larger than the error of the other suspensions. This meant that the numbers of green and red dyed cells varied largely, and the chances for making pairs were decreased. Because the number of cells was too large, many cells could remain stuck in the microchannel. Thus, in this study, we judged the best concentration of the cell suspension was 2.57 × 10^4^ cells/mL.

### 3.2. Electrofusion and Substance Transport between the Cell and the Giant Liposome

**Figure 7:**
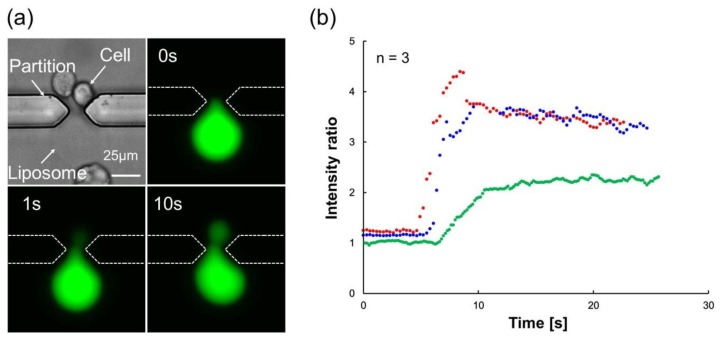
(**a**) Bright field image of the cell and the giant liposome before AC voltage application and the fluorescent image after DC voltage application at 0 s, 1 s, and 10 s. Electrofusion and Calcein transport occurred between a cell and a giant liposome. (**b**) Transition intracellular luminance normalized with background luminance.

Because studies observing substance transport between a liposome and a cell are rare, the observation of transport is important. Figure 7a and Video S1 show the bright field image of the cell and the giant liposome before AC voltage application and the fluorescent image after DC voltage application at 0 s, 1 s, and 10 s. Introduction of the fluorescent inner solution (Calcein) of the liposome into the cell by electrofusion was observed. Generally, Calcein does not pass through the cell membrane. However, in this observation, Calcein was transported from liposomes to cells. Thus, we considered that a membrane fusion occurred between liposomes and cells. As shown in Figure 7b, increase of intracellular luminance after DC voltage application was observed. The percentages of transported substance amount were 22.4 ± 0.1%, 10.3 ± 0.4% and 9.1 ± 0.1% (*n* = 3). The shrinkage of liposome was not observed. This indicated that the substance of the liposome and the cell could be changed by diffusion. Our fusion success ratio (transport success ratio) was 8%. There have been many studies which tried to fuse cells in micro devices by electrofusion [17,18,19,20], and our fusion success ratio was lower than the ratios of other studies which described cell–cell electrofusion (50% [18], 80–95% [19] and 78–90% [20]). We consider that perhaps the success ratio of cell–liposome fusion may be lower than the success ratio of cell–cell fusion, because the success ratios of our study (8%) and another study (20% [14]) which fused liposome and cell were lower than ratios in these studies. Even so, our cell–liposome fusion success ratio was smaller than that of the other study (20%) [14]. The low efficiency of the fusion success ratio could be due to two reasons. The first reason was the limited field of view. In this study, we could observe only two gaps in the microscope field and perhaps fusions happened in other gaps. However, it was difficult to observe other places because the observation magnitude was high and the observation field was small. We inserted the device into the cell suspension 30–49 times, and also applied the AC voltage the same number of times for making pairs at each observation. We confirmed 50 pairs of liposome and cell after all application of AC voltage. Then, the DC voltage for fusion was applied less than 49 times. Because we could observe two gaps in the microscope field, the number of times of confirmation for pairs was larger than the number of times for application of AC voltage and the number of times for applying DC voltage. After applying DC voltage, the fusion of one pair was observed two times and fusion of two pairs was observed one time. Thus, we assumed a fusion ratio of 8%. However, we could have missed observing fusions occurred in other gaps. Thus, this fusion ratio might not necessarily represent the actual number of fusions that occurred. The second reason was the concentration of fusion buffer. When the concentration of fusion buffer was low, the efficiency of fusion was also low. The diffusion coefficient and the time constant were calculated as 137 ± 18 μm^2^/s and 1.06 ± 0.10 s, respectively, as shown in Table 1. Standard error was calculated for three cells for which we could observe luminance transition.

Improvement in the cell membrane perforation efficiency and fusion efficiency with the giant liposome could be expected by applying the DC voltage in a fusion buffer of lower concentration. However, cells could burst if a too hypotonic buffer solution is used, thus we adopted the concentration of 110 mM, at which cell bursting was not observed in this study. Since the total luminance inside the cell and the liposome was preserved before and after the DC voltage application, successful transport of the fluorescent substance (Calcein) into the cell without leakage outside the cell and the liposome was indicated. Moreover, at that time the cell was not fused with the liposome completely because of the partition and the cell maintained its shape. In this study, it was difficult to culture fused cells because fused cells were washed away by the flow applied when changing the buffer solution for electrofusion to the culture solution. Then, we could not make follow-up observations on culturing of the fused cells. This means that we lost sight of the cells which we had to observe. Therefore, we did not carry out culturing of fused cells. However, it is important to observe culturing cells after fusion. Thus, in the near future, we intend to modify the device.

The diameter of liposomes used in this study was around 30–40 µm because we got liposomes more efficiently in this condition. We expect it is possible to decrease the diameter of the liposomes because another study succeeded in adjusting the diameter of giant liposomes [11], and we might also be able to fuse these liposomes. Thus, if we can control the diameter of the liposome and transport substances more surely, transport of a precise amount of substance can become a reality. After finishing the electrofusion, by applying flow in the micro channel, cells in which substances were transported by a liposome and liposomes can be separated. We propose the possibility that substances can be transported to the cells with less damage of the cell membrane.

The measured diffusion coefficient was one order of magnitude higher than that of the fluorescent substance in the cell [21]. When considering the diffusion of particles in liquid, the Stokes–Einstein equation can be expressed as Equation (3):

(3) $$D=\frac{k_{B}T}{6\pi \eta r}$$

where *D* is the diffusion coefficient of the particle, *K_B_* is Boltzmann’s constant, *T* is the absolute temperature, *η* is the viscosity of solvent, and *r* is the radius of the particle [22]. Equation (3) indicates that the diffusion coefficient of the fluorescent substance is inversely proportional to the increase in intracellular viscosity. Figure S1 shows the bright field image of cells in the 110 mM, 310 mM, and 510 mM fusion buffers. The cells shrank in the 310 mM and 510 mM hypertonic fusion buffers. On the other hand, the cells were swollen in the 110 mM hypotonic fusion buffer, and that indicated inflow of pure water into the cells occurred due to osmotic pressure. Since the intracellular viscosity decreased below the usual state due to the inflow of pure water, we considered that the diffusion coefficient of the Calcein in the 110 mM fusion buffer was increased.

Our demonstrated method can transport a small amount of substrate from a liposome to a cell precisely without leakage. Thus, this method can offer a single-cell level cure. If a small amount of a drug is introduced to inner cells precisely, regional and specific cures may become a reality.

## 4. Conclusions

We successfully carried out an electrofusion and a small amount of substance transport between a C2C12 cell that held its shape and a giant liposome without leakage of liquid. Additionally, we also successfully observed the transport of substance from the liposome to cells in detail. To observe electrofusion from the moment of DC voltage application, we fabricated the microfluidic device and optimized the suspension concentrations of cells and giant liposomes for efficient pair formation in the microchannel. Electrofusion and substance transport between a cell and a giant liposome was observed in the fabricated microfluidic device. The intracellular viscosity was decreased by inflow of pure water in hypotonic buffer based on the measurement results of diffusion coefficient of the fluorescent substance (Calcein). Only a few studies have observed the substance transport between a liposome and a cell. Thus, the knowledge presented in this report is important for the field of liposome research. In the future, we will consider transport of substances such as micro- and nanostructures [14] or fabricate micro structures [12] with the proposed method. The present results show the validity of the giant liposome as an accurate substance transport capsule for future research requiring single cell analysis which needs a micro-, nano- or pico-liter order fluidic device which can accurately control the amount of substances.

## Figures and Tables

**Table 1:** The diffusion coefficient, the time constant and the fusion ratio were derived by the observation of the fluorescent inner solution of the liposome into the cell by electrofusion.

Parameter	Value
Diffusion coefficient (µm^2^/s)	137 ± 18
Time constant (s)	1.06 ± 0.10
Fusion ratio (%)	8

## Supplementary Materials

The following are available online at http://www.mdpi.com/2072-666X/9/7/331/s1, Figure S1: Bright field image of cells in the 110 mM, 310 mM, and 510 mM fusion buffer, and Video S1: The fluorescent image after DC voltage application at 5 s.
