# Supplementary material for: A Closed System for Pico-Liter Order Substance Transport from a Giant Liposome to a Cell
Source: Micromachines (Basel). 2018 Jul 2;9(7):331. doi: 10.3390/mi9070331 (PMC6082288; doi:10.3390/mi9070331)
Supplement: Supplementary file 1 [file micromachines-09-00331-s001.zip › micromachines-294417-SI.pdf]

Supplementary Material: A Closed System for Pico-Liter Order Substance Transport from a Giant Liposome to a Cell

Shohei Miyakawa, Kaoru Uesugi and Keisuke Morishima

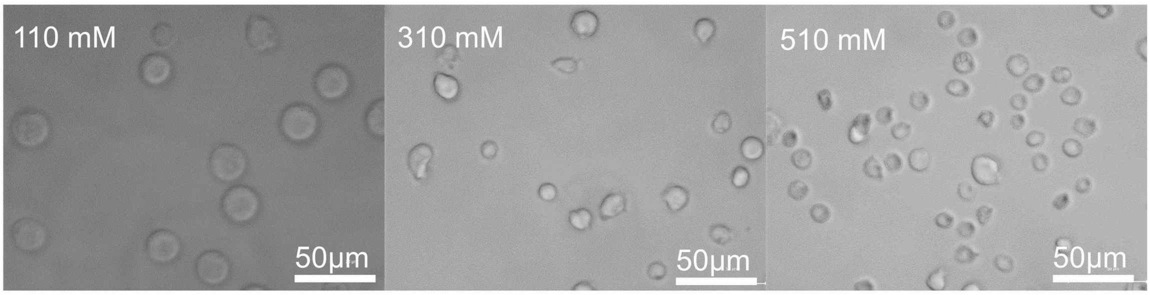

Figure S1. Bright field image of cells in the 110 mM, 310 mM, and 510 mM fusion buffer.
